# 2125 What genes are involved in the brain food reward circuitry: Findings from a large candidate gene analysis

**DOI:** 10.1017/cts.2018.149

**Published:** 2018-11-21

**Authors:** Ying Meng, Susan W. Groth, Joyce A. Smith, Harriet Kitzman

**Affiliations:** 1 University of Rochester Medical Center; 2 University of Rochester School of Nursing

## Abstract

OBJECTIVES/SPECIFIC AIMS: The food reward circuitry regulates hedonic eating especially in relation to palatable hypercaloric foods, which can lead to chronic overeating and consequent overweight and obesity. Evidence supports that there is considerable overlap within the brain reward circuitry between palatable hypercaloric food intake and substance addiction. The goal of this study was to identify associations between addiction-related genes and body mass index. We hypothesized that addiction-related genes potentially participate in the food reward circuitry if they are associated with obesity traits. METHODS/STUDY POPULATION: A secondary analysis was conducted with 1093 African American adolescents and young adults from the New Mother’s Study. Anthropometric, genetic, demographic and lifestyle measurements were available at the 18-year follow-up assessments. A total of 1350 single nucleotide polymorphisms mapped to 127 addiction-related genes were assessed. A total of 186 ancestry informative markers were used to adjust for population stratification. Generalized estimating equation models were used to identify genetic associations, including additive, dominant, and recessive models, and control for correlations within families. RESULTS/ANTICIPATED RESULTS: The participants ranged from 15 to 23 years of age. Of them, 42.7% were overweight or obese. Significant associations with body mass index were identified for 13 single nucleotide polymorphisms mapped to 11 addiction-related genes, including LEP (*p* 0.027–<0.001). Most of these genes are involved in dopaminergic, opioidergic, serotonergic pathways, and stress. DISCUSSION/SIGNIFICANCE OF IMPACT: Our results support the role of dopaminergic and opioidergic pathways in the food reward circuitry, and suggest a potential involvement of serotonergic pathways and genes related to stress in the food reward circuitry. Further investigation of the identified genes will facilitate delineation and understanding of the brain food reward system and its relationship with obesity.

